# Bis(μ-quinolin-8-olato)-κ^3^ *N*,*O*:*O*;κ^3^ *O*:*N*,*O*-bis­[chloridomethyl­phenyl­tin(IV)]

**DOI:** 10.1107/S1600536810027765

**Published:** 2010-07-17

**Authors:** Maryam Vafaee, Mostafa M. Amini, Seik Weng Ng

**Affiliations:** a Department of Chemistry, General Campus, Shahid Beheshti University, Tehran 1983963113, Iran; b Department of Chemistry, University of Malaya, 50603 Kuala Lumpur, Malaysia

## Abstract

The Sn^IV^ atom in the centrosymmetric dinculear title compound, [Sn_2_(CH_3_)_2_(C_6_H_5_)_2_(C_9_H_6_NO)_2_Cl_2_], shows a *trans*-C_2_SnNO_2_Cl distorted octa­hedral coordination [C–Sn–C = 157.83 (8)°]. The quinolin-8-olate anion chelates to the Sn atom; its O atom also binds to the inversion-related Sn atom, forming the dinuclear compound. In the crystal structure, weak inter­molecular C—H⋯Cl hydrogen bonding links the mol­ecules, forming supra­molecular chains running along [100].

## Related literature

For related structures, see: Ng *et al.* (1989 ▶); Shi & Hu (1987 ▶).
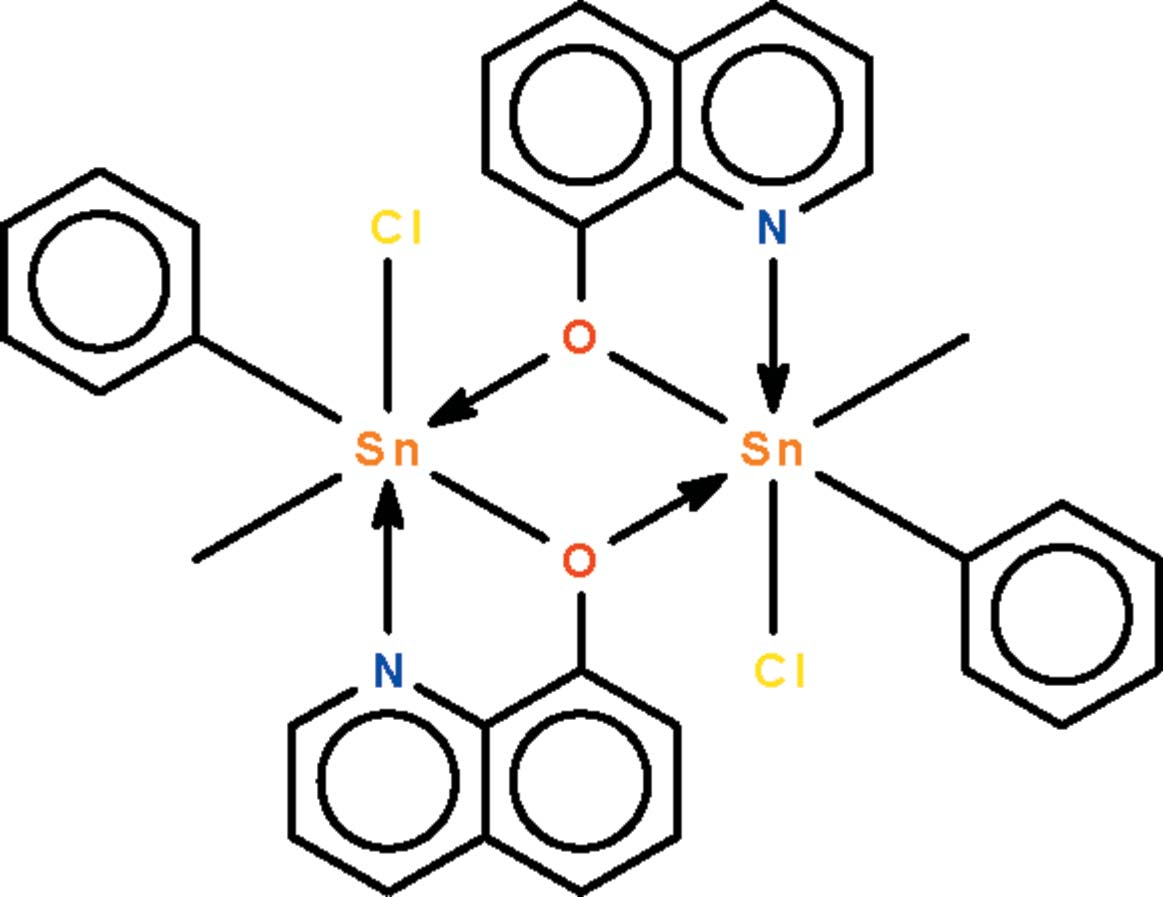


## Experimental

### 

#### Crystal data

[Sn_2_(CH_3_)_2_(C_6_H_5_)_2_(C_9_H_6_NO)_2_Cl_2_]
*M* *_r_* = 780.84
Monoclinic, 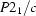

*a* = 7.9967 (5) Å
*b* = 17.8081 (10) Å
*c* = 10.1623 (6) Å
β = 95.232 (1)°
*V* = 1441.14 (15) Å^3^
*Z* = 2
Mo *K*α radiation
μ = 1.95 mm^−1^
*T* = 100 K
0.30 × 0.20 × 0.10 mm

#### Data collection

Bruker SMART APEX diffractometer
Absorption correction: multi-scan (*SADABS*; Sheldrick, 1996 ▶) *T*_min_ = 0.592, *T*_max_ = 0.829
9127 measured reflections
3245 independent reflections
3088 reflections with *I* > 2σ(*I*)
*R*_int_ = 0.017

#### Refinement

*R*[*F* ^2^ > 2σ(*F* ^2^)] = 0.018
*wR*(*F* ^2^) = 0.047
*S* = 1.09
3245 reflections
182 parameters
H-atom parameters constrained
Δρ_max_ = 0.43 e Å^−3^
Δρ_min_ = −0.52 e Å^−3^

### 

Data collection: *APEX2* (Bruker, 2009 ▶); cell refinement: *SAINT* (Bruker, 2009 ▶); data reduction: *SAINT*; program(s) used to solve structure: *SHELXS97* (Sheldrick, 2008 ▶); program(s) used to refine structure: *SHELXL97* (Sheldrick, 2008 ▶); molecular graphics: *X-SEED* (Barbour, 2001 ▶); software used to prepare material for publication: *publCIF* (Westrip, 2010 ▶).

## Supplementary Material

Crystal structure: contains datablocks global, I. DOI: 10.1107/S1600536810027765/xu2799sup1.cif

Structure factors: contains datablocks I. DOI: 10.1107/S1600536810027765/xu2799Isup2.hkl

Additional supplementary materials:  crystallographic information; 3D view; checkCIF report

## Figures and Tables

**Table 1:** Hydrogen-bond geometry (Å, °)

*D*—H⋯*A*	*D*—H	H⋯*A*	*D*⋯*A*	*D*—H⋯*A*
C6—H6⋯Cl1^i^	0.95	2.76	3.710 (2)	174

Symmetry code: (i) 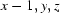.

**Fig. 1.**
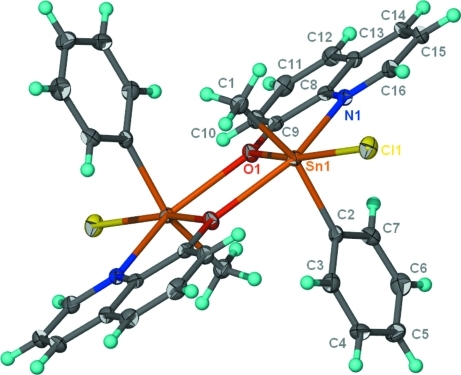
Thermal ellipsoid plot (Barbour, 2001) of [SnCl(CH3)(C6H5)(C9H6NO)]2 at the 70% probability level. Hydrogen atoms are drawn as spheres of arbitrary radius.

## supplementary crystallographic information

### Comment

The anion of 8-hydroxyquinoline is known to chelate to tin in organotin(IV) quinolinolates; however, for the chloroorganotin quinolinates, the chlorine atom sometimes participates in weak intermolecular bridging. In chloridoodiethyl(quinolin-8-olato)tin, the carbon–tin–carbon angle is opened to 140.9 (3) ° owing to a tin···chlorine contact of 3.690 (2) Å (Shi & Hu, 1987). With the bis(2-carbomethoxyethyl) analog, the tin atom is six-coordinate owing to an intramolecular bond with the oxygen atom of the organo radical (Ng *et al.*, 1989). The chloridomethylphenyltin analog exists as a centrosymmetric dimer in which the quinolin-8-olate anion *N*,*O*-chelates to the tin atom (Fig. 1). However, its oxygen atom also binds to the inversion-related tin atom so that bridging by the chlorine atom is precluded for the *trans*-C_2_SnNO_2_Cl octahedral dinuclear molecule. Intermolecular weak C—H···Cl hydrogen bonding links the molecules to form the one dimensional supra-molecular chain in the crystal structure (Table 1).

### Experimental

Methylphenyltin dichloride (0.35 g, 1 mmol) and 8-hydroxyquinoline (0.15 g, 1 mmol) were dissolved in methanol (10 ml) to give a faint yellow solution. The solution was set aside for the growth of crystals over a few days. Slow evaporation of methanol furnished crystals.

### Refinement

Hydrogen atoms were placed in calculated positions (C–H 0.95–0.98 Å) and were included in the refinement in the riding model approximation, with *U*(H) set to 1.2–1.5*U*_eq_(C).

The final difference Fourier map had a peak in the vicinity of Sn1.

### Crystal data

**Table:**

[Sn_2_(CH_3_)_2_(C_6_H_5_)_2_(C_9_H_6_NO)_2_Cl_2_]	*F*(000) = 768
*M**_r_* = 780.84	*D*_x_ = 1.799 Mg m^−^^3^
Monoclinic, *P*2_1_/*c*	Mo *K*α radiation, λ = 0.71073 Å
Hall symbol: -P 2ybc	Cell parameters from 6739 reflections
*a* = 7.9967 (5) Å	θ = 2.3–28.3°
*b* = 17.8081 (10) Å	µ = 1.95 mm^−^^1^
*c* = 10.1623 (6) Å	*T* = 100 K
β = 95.232 (1)°	Block, yellow
*V* = 1441.14 (15) Å^3^	0.30 × 0.20 × 0.10 mm
*Z* = 2	

### Data collection

**Table:**

Bruker SMART APEX diffractometer	3245 independent reflections
Radiation source: fine-focus sealed tube	3088 reflections with *I* > 2σ(*I*)
graphite	*R*_int_ = 0.017
ω scans	θ_max_ = 27.5°, θ_min_ = 2.3°
Absorption correction: multi-scan (*SADABS*; Sheldrick, 1996)	*h* = −10→6
*T*_min_ = 0.592, *T*_max_ = 0.829	*k* = −23→23
9127 measured reflections	*l* = −12→12

### Refinement

**Table:**

Refinement on *F*^2^	Primary atom site location: structure-invariant direct methods
Least-squares matrix: full	Secondary atom site location: difference Fourier map
*R*[*F*^2^ > 2σ(*F*^2^)] = 0.018	Hydrogen site location: inferred from neighbouring sites
*wR*(*F*^2^) = 0.047	H-atom parameters constrained
*S* = 1.09	*w* = 1/[σ^2^(*F*_o_^2^) + (0.021*P*)^2^ + 1.1915*P*] where *P* = (*F*_o_^2^ + 2*F*_c_^2^)/3
3245 reflections	(Δ/σ)_max_ = 0.001
182 parameters	Δρ_max_ = 0.43 e Å^−^^3^
0 restraints	Δρ_min_ = −0.52 e Å^−^^3^

### Fractional atomic coordinates and isotropic or equivalent isotropic displacement parameters (Å^2^)

**Table:**

	*x*	*y*	*z*	*U*_iso_*/*U*_eq_	
Sn1	0.569331 (15)	0.585876 (6)	0.601796 (12)	0.01090 (5)	
Cl1	0.70000 (6)	0.71504 (2)	0.65698 (5)	0.01757 (10)	
O1	0.42527 (16)	0.48265 (7)	0.60854 (13)	0.0135 (3)	
N1	0.4714 (2)	0.58459 (8)	0.80183 (16)	0.0121 (3)	
C1	0.8095 (2)	0.53633 (10)	0.6389 (2)	0.0167 (4)	
H1A	0.7981	0.4816	0.6403	0.025*	
H1B	0.8618	0.5536	0.7245	0.025*	
H1C	0.8800	0.5508	0.5691	0.025*	
C2	0.3678 (2)	0.64342 (9)	0.49452 (19)	0.0130 (4)	
C3	0.3885 (3)	0.67691 (11)	0.3733 (2)	0.0169 (4)	
H3	0.4928	0.6723	0.3361	0.020*	
C4	0.2584 (3)	0.71696 (11)	0.3060 (2)	0.0195 (4)	
H4	0.2734	0.7388	0.2226	0.023*	
C5	0.1060 (3)	0.72510 (11)	0.3607 (2)	0.0207 (4)	
H5	0.0176	0.7533	0.3156	0.025*	
C6	0.0837 (3)	0.69195 (11)	0.4814 (2)	0.0206 (4)	
H6	−0.0198	0.6975	0.5194	0.025*	
C7	0.2136 (2)	0.65061 (10)	0.5464 (2)	0.0164 (4)	
H7	0.1967	0.6268	0.6279	0.020*	
C8	0.3633 (2)	0.52614 (10)	0.81865 (18)	0.0119 (3)	
C9	0.3396 (2)	0.47262 (10)	0.71380 (18)	0.0124 (3)	
C10	0.2317 (2)	0.41329 (10)	0.7296 (2)	0.0147 (4)	
H10	0.2124	0.3772	0.6611	0.018*	
C11	0.1502 (2)	0.40557 (10)	0.8459 (2)	0.0173 (4)	
H11	0.0772	0.3641	0.8544	0.021*	
C12	0.1735 (2)	0.45627 (10)	0.9470 (2)	0.0162 (4)	
H12	0.1177	0.4497	1.0248	0.019*	
C13	0.2812 (2)	0.51873 (10)	0.93531 (19)	0.0133 (4)	
C14	0.3148 (3)	0.57403 (11)	1.0345 (2)	0.0160 (4)	
H14	0.2616	0.5713	1.1142	0.019*	
C15	0.4242 (2)	0.63159 (10)	1.0154 (2)	0.0165 (4)	
H15	0.4471	0.6688	1.0816	0.020*	
C16	0.5016 (2)	0.63503 (10)	0.89730 (19)	0.0146 (4)	
H16	0.5781	0.6747	0.8851	0.018*	

### Atomic displacement parameters (Å^2^)

**Table:**

	*U*^11^	*U*^22^	*U*^33^	*U*^12^	*U*^13^	*U*^23^
Sn1	0.01182 (7)	0.01028 (7)	0.01071 (8)	0.00014 (4)	0.00166 (5)	0.00028 (4)
Cl1	0.0161 (2)	0.01251 (19)	0.0241 (3)	−0.00307 (15)	0.00144 (18)	−0.00177 (16)
O1	0.0167 (6)	0.0120 (6)	0.0123 (7)	−0.0016 (5)	0.0041 (5)	−0.0008 (5)
N1	0.0147 (8)	0.0107 (7)	0.0110 (8)	0.0009 (5)	0.0018 (6)	0.0001 (5)
C1	0.0155 (9)	0.0161 (8)	0.0185 (10)	0.0022 (7)	0.0006 (7)	−0.0006 (7)
C2	0.0149 (9)	0.0093 (7)	0.0144 (9)	−0.0004 (6)	−0.0006 (7)	−0.0027 (6)
C3	0.0181 (9)	0.0157 (8)	0.0170 (10)	0.0016 (7)	0.0027 (8)	−0.0005 (7)
C4	0.0269 (11)	0.0179 (9)	0.0133 (10)	0.0027 (8)	−0.0012 (8)	0.0020 (7)
C5	0.0206 (10)	0.0178 (9)	0.0221 (11)	0.0035 (7)	−0.0064 (8)	−0.0015 (8)
C6	0.0146 (9)	0.0228 (10)	0.0242 (11)	0.0002 (7)	0.0009 (8)	−0.0043 (8)
C7	0.0179 (9)	0.0182 (9)	0.0131 (10)	−0.0013 (7)	0.0014 (7)	−0.0010 (7)
C8	0.0128 (8)	0.0120 (8)	0.0107 (9)	0.0014 (6)	0.0005 (7)	0.0015 (6)
C9	0.0125 (8)	0.0125 (8)	0.0121 (9)	0.0027 (6)	0.0007 (7)	0.0012 (7)
C10	0.0154 (9)	0.0127 (8)	0.0159 (10)	−0.0003 (6)	0.0006 (7)	−0.0013 (7)
C11	0.0147 (9)	0.0143 (8)	0.0232 (11)	−0.0018 (7)	0.0030 (8)	0.0026 (7)
C12	0.0149 (9)	0.0178 (9)	0.0169 (10)	0.0018 (7)	0.0057 (7)	0.0032 (7)
C13	0.0135 (9)	0.0145 (8)	0.0122 (9)	0.0030 (6)	0.0019 (7)	0.0002 (7)
C14	0.0207 (10)	0.0196 (9)	0.0084 (9)	0.0042 (7)	0.0045 (7)	−0.0001 (7)
C15	0.0208 (10)	0.0157 (8)	0.0128 (10)	0.0035 (7)	−0.0001 (8)	−0.0033 (7)
C16	0.0155 (9)	0.0132 (8)	0.0148 (10)	0.0007 (7)	−0.0003 (7)	−0.0010 (7)

### Geometric parameters (Å, °)

**Table:**

Sn1—C1	2.1162 (19)	C5—H5	0.9500
Sn1—C2	2.1248 (18)	C6—C7	1.390 (3)
Sn1—O1	2.1739 (13)	C6—H6	0.9500
Sn1—O1^i^	2.4651 (13)	C7—H7	0.9500
Sn1—N1	2.2442 (16)	C8—C13	1.413 (3)
Sn1—Cl1	2.5672 (5)	C8—C9	1.429 (2)
O1—C9	1.334 (2)	C9—C10	1.383 (3)
O1—Sn1^i^	2.4651 (13)	C10—C11	1.408 (3)
N1—C16	1.328 (2)	C10—H10	0.9500
N1—C8	1.373 (2)	C11—C12	1.367 (3)
C1—H1A	0.9800	C11—H11	0.9500
C1—H1B	0.9800	C12—C13	1.419 (3)
C1—H1C	0.9800	C12—H12	0.9500
C2—C3	1.392 (3)	C13—C14	1.417 (3)
C2—C7	1.391 (3)	C14—C15	1.373 (3)
C3—C4	1.388 (3)	C14—H14	0.9500
C3—H3	0.9500	C15—C16	1.401 (3)
C4—C5	1.393 (3)	C15—H15	0.9500
C4—H4	0.9500	C16—H16	0.9500
C5—C6	1.387 (3)		
			
C1—Sn1—C2	157.83 (8)	C6—C5—C4	119.75 (19)
C1—Sn1—O1	96.71 (6)	C6—C5—H5	120.1
C2—Sn1—O1	92.56 (6)	C4—C5—H5	120.1
C1—Sn1—N1	102.73 (7)	C5—C6—C7	119.69 (19)
C2—Sn1—N1	99.13 (7)	C5—C6—H6	120.2
O1—Sn1—N1	74.52 (5)	C7—C6—H6	120.2
C1—Sn1—O1^i^	81.99 (6)	C2—C7—C6	121.21 (19)
C2—Sn1—O1^i^	82.31 (6)	C2—C7—H7	119.4
O1—Sn1—O1^i^	70.12 (5)	C6—C7—H7	119.4
N1—Sn1—O1^i^	144.64 (5)	N1—C8—C13	121.40 (16)
C1—Sn1—Cl1	89.42 (5)	N1—C8—C9	117.06 (16)
C2—Sn1—Cl1	87.39 (5)	C13—C8—C9	121.54 (16)
O1—Sn1—Cl1	163.16 (4)	O1—C9—C10	124.56 (17)
N1—Sn1—Cl1	88.85 (4)	O1—C9—C8	117.74 (16)
O1^i^—Sn1—Cl1	126.44 (3)	C10—C9—C8	117.69 (17)
C9—O1—Sn1	116.80 (11)	C9—C10—C11	120.93 (18)
C9—O1—Sn1^i^	132.84 (11)	C9—C10—H10	119.5
Sn1—O1—Sn1^i^	109.88 (5)	C11—C10—H10	119.5
C16—N1—C8	119.75 (17)	C12—C11—C10	121.63 (18)
C16—N1—Sn1	126.85 (13)	C12—C11—H11	119.2
C8—N1—Sn1	113.33 (12)	C10—C11—H11	119.2
Sn1—C1—H1A	109.5	C11—C12—C13	119.79 (18)
Sn1—C1—H1B	109.5	C11—C12—H12	120.1
H1A—C1—H1B	109.5	C13—C12—H12	120.1
Sn1—C1—H1C	109.5	C14—C13—C8	117.37 (17)
H1A—C1—H1C	109.5	C14—C13—C12	124.21 (18)
H1B—C1—H1C	109.5	C8—C13—C12	118.41 (17)
C3—C2—C7	118.50 (18)	C15—C14—C13	119.99 (18)
C3—C2—Sn1	121.01 (14)	C15—C14—H14	120.0
C7—C2—Sn1	120.46 (14)	C13—C14—H14	120.0
C2—C3—C4	120.78 (19)	C14—C15—C16	119.41 (18)
C2—C3—H3	119.6	C14—C15—H15	120.3
C4—C3—H3	119.6	C16—C15—H15	120.3
C5—C4—C3	120.03 (19)	N1—C16—C15	122.08 (17)
C5—C4—H4	120.0	N1—C16—H16	119.0
C3—C4—H4	120.0	C15—C16—H16	119.0
			
C1—Sn1—O1—C9	107.94 (13)	C3—C4—C5—C6	−1.1 (3)
C2—Sn1—O1—C9	−92.24 (13)	C4—C5—C6—C7	−0.2 (3)
N1—Sn1—O1—C9	6.53 (12)	C3—C2—C7—C6	−1.8 (3)
O1^i^—Sn1—O1—C9	−173.12 (15)	Sn1—C2—C7—C6	176.04 (14)
Cl1—Sn1—O1—C9	−2.8 (2)	C5—C6—C7—C2	1.7 (3)
C1—Sn1—O1—Sn1^i^	−78.95 (7)	C16—N1—C8—C13	0.7 (3)
C2—Sn1—O1—Sn1^i^	80.88 (7)	Sn1—N1—C8—C13	−176.16 (13)
N1—Sn1—O1—Sn1^i^	179.65 (7)	C16—N1—C8—C9	−178.36 (16)
O1^i^—Sn1—O1—Sn1^i^	0.0	Sn1—N1—C8—C9	4.7 (2)
Cl1—Sn1—O1—Sn1^i^	170.35 (8)	Sn1—O1—C9—C10	174.90 (14)
C1—Sn1—N1—C16	83.99 (16)	Sn1^i^—O1—C9—C10	3.7 (3)
C2—Sn1—N1—C16	−92.33 (16)	Sn1—O1—C9—C8	−6.4 (2)
O1—Sn1—N1—C16	177.53 (16)	Sn1^i^—O1—C9—C8	−177.52 (11)
O1^i^—Sn1—N1—C16	178.10 (13)	N1—C8—C9—O1	0.9 (2)
Cl1—Sn1—N1—C16	−5.16 (15)	C13—C8—C9—O1	−178.23 (16)
C1—Sn1—N1—C8	−99.37 (13)	N1—C8—C9—C10	179.70 (16)
C2—Sn1—N1—C8	84.31 (13)	C13—C8—C9—C10	0.6 (3)
O1—Sn1—N1—C8	−5.84 (12)	O1—C9—C10—C11	177.91 (17)
O1^i^—Sn1—N1—C8	−5.27 (17)	C8—C9—C10—C11	−0.8 (3)
Cl1—Sn1—N1—C8	171.48 (12)	C9—C10—C11—C12	0.3 (3)
C1—Sn1—C2—C3	−8.7 (3)	C10—C11—C12—C13	0.4 (3)
O1—Sn1—C2—C3	−123.54 (15)	N1—C8—C13—C14	0.0 (3)
N1—Sn1—C2—C3	161.73 (14)	C9—C8—C13—C14	179.06 (17)
O1^i^—Sn1—C2—C3	−53.99 (15)	N1—C8—C13—C12	−178.93 (16)
Cl1—Sn1—C2—C3	73.32 (14)	C9—C8—C13—C12	0.1 (3)
C1—Sn1—C2—C7	173.51 (16)	C11—C12—C13—C14	−179.50 (19)
O1—Sn1—C2—C7	58.69 (15)	C11—C12—C13—C8	−0.6 (3)
N1—Sn1—C2—C7	−16.04 (15)	C8—C13—C14—C15	−0.4 (3)
O1^i^—Sn1—C2—C7	128.23 (15)	C12—C13—C14—C15	178.46 (18)
Cl1—Sn1—C2—C7	−104.46 (14)	C13—C14—C15—C16	0.1 (3)
C7—C2—C3—C4	0.4 (3)	C8—N1—C16—C15	−1.1 (3)
Sn1—C2—C3—C4	−177.45 (14)	Sn1—N1—C16—C15	175.35 (13)
C2—C3—C4—C5	1.1 (3)	C14—C15—C16—N1	0.7 (3)

Symmetry codes: (i) −*x*+1, −*y*+1, −*z*+1.

### Hydrogen-bond geometry (Å, °)

**Table:**

*D*—H···*A*	*D*—H	H···*A*	*D*···*A*	*D*—H···*A*
C6—H6···Cl1^ii^	0.95	2.76	3.710 (2)	174

Symmetry codes: (ii) *x*−1, *y*, *z*.
